# Special Issue “Mosquito-Borne Virus Ecology”

**DOI:** 10.3390/v14020357

**Published:** 2022-02-09

**Authors:** Jonas Schmidt-Chanasit, Eric Agboli, Hanna Jöst

**Affiliations:** 1 Bernhard Nocht Institute for Tropical Medicine, WHO Collaborating Centre for Arbovirus and Haemorrhagic Fever Reference and Research, 20359 Hamburg, Germany; jonassi@gmx.de (J.S.-C.); agboli@bnitm.de (E.A.); 2 Faculty of Mathematics, Informatics and Natural Sciences, University of Hamburg, 22609 Hamburg, Germany

Mosquito-borne viruses (MBVs), also known as moboviruses, are associated mainly with mosquitoes and are able to infect humans and other vertebrates. Globally, diseases caused by MBVs such as dengue virus and Zika virus are growing public health concerns. However, the circulation of MBVs are mainly driven by the mosquito vectors. A transmission from human to human is rare and normally does not contribute to the circulation of MBVs. Several species of mosquitoes are involved in the transmission and circulation of these viruses. The main vector of concern is the *Aedes aegypti* mosquito, which is known to spread major devastating MBVs of public health concern. Ecological factors such as climate change, urbanization, and land-use drive the maintenance and potential circulation of MBVs’. A better understanding of mosquito vectors, their ecology, and their role in the transmission of MBVs can support forecasts regarding potential outbreaks and can improve sustainable vector control strategies and their implementations. This implies that a deeper knowledge of the ecological factors that may trigger MBV epidemics are of utmost importance.

The aim of this Special Issue, “Mosquito-Borne Virus Ecology”, was to compile scientific data on MBVs that are currently of public health importance and the effect of ecological factors influencing the occurrence and transmission risks of these viruses. This Special Issue has some interesting contributions to science in relation to the geographical distribution of MBVs, vector competence, the effect of temperature, and mosquito vector surveillance [1,2,3,4,5,6,7,8,9,10,11]. It includes both original and review articles that provide important contributions to the scientific literature. The distribution of mosquito-associated viruses in West Africa was analyzed, which provides a general overview of mosquito vectors and MBVs recorded in the West African region [3]. Different studies also reported the vector competence of different mosquito vectors for certain MBVs [4,5,9,10,11]. The competence of a mosquito vector for a particular MBV depends on the intrinsic and extrinsic characteristics of both the vector and the target virus. Most infection studies are conducted in laboratories using static incubation temperatures, which may not be the same temperatures as those to which mosquitoes are exposed daily, compromising the applicability of the resultant data to real-world scenarios [5]. Another study reported that insect-specific viruses (ISVs) have the potential to affect the replication of MBVs, and thus, have potential to be used as a tool in biological vector control [12]. A novel Wiesbaden virus, which is an ISV, was reported to have an effect that boosts chikungunya virus transmission [4].

Despite these significant contributions to our understanding of MBVs, future studies are needed to accurately understand MBVs; their impact on mosquito vector ecology; and the interaction between MBVs, mosquito vector, and the environment. Finally, we are grateful to the authors and reviewers of this Special Issue who provided their expertise on relevant scientific information from their respective specialties. We sincerely hope that future calls for studies and peer-review will be met with the same energy they provided during this work.
